# Genome assemblies of three closely related leaf beetle species (*Galerucella* spp.)

**DOI:** 10.1093/g3journal/jkab214

**Published:** 2021-06-22

**Authors:** Xuyue Yang, Tanja Slotte, Jacques Dainat, Peter A Hambäck

**Affiliations:** 1 Department of Ecology, Environment and Plant Sciences, Stockholm University, Stockholm 10691, Sweden; 2 Department of Medical Biochemistry Microbiology and Genomics, National Bioinformatics Infrastructure Sweden, Science for Life Laboratory, Uppsala University, Uppsala 75237, Sweden

**Keywords:** *Galerucella calmariensis*, *Galerucella pusilla*, *Galerucella tenella*, Coleoptera, leaf beetle

## Abstract

*Galerucella* (Coleoptera: Chrysomelidae) is a leaf beetle genus that has been extensively used for ecological and evolutionary studies. It has also been used as biological control agent against invading purple loosestrife in North America, with large effects on biodiversity. Here, we report genome assembly and annotation of three closely related *Galerucella* species: *G. calmariensis*, *G. pusilla*, and *G. tenella*. The three assemblies have a genome size ranging from 460 to 588 Mbp, with N50 from 31,588 to 79,674 kbp, containing 29,202 to 40,929 scaffolds. Using an *ab initio* evidence-driven approach, 30,302 to 33,794 protein-coding genes were identified and functionally annotated. These draft genomes will contribute to the understanding of host-parasitoid interactions, evolutionary comparisons of leaf beetle species and future population genomics studies.

## Introduction

*Galerucella* (Coleoptera: Chrysomelidae) is a leaf beetle genus that is distributed worldwide except in the Neotropics (Thomas *et al.* 2002). Some species have been used as biological control agents against invasive plants, and the host specificity and environmental impact of these species have attracted broad interest. The most common application is the introduction of *G. calmariensis* and *G. pusilla* from Europe to North America against the invasive wetland plant purple loosestrife (*Lythrum salicaria*). Since 1992, releases of *Galerucella* populations have been made in many states in the USA and the colonization appears to have been successful, leading to a dramatic decrease of *L. salicaria* populations (Blossey *et al.* 1994; Landis *et al.* 2003; McAvoy *et al.* 2016).

In addition to its application in biological control, *Galerucella* spp. has been widely investigated in both ecological (Pappers *et al.* 2002; Tanaka and Nakasuji 2002; Hori *et al.* 2006; Fors *et al.* 2016) and evolutionary (Ikonen *et al.* 2003; Stenberg and Axelsson 2008; Yang *et al.* 2020) studies. In particular, *Galerucella* spp. has been used to study ecological and evolutionary consequences of host-parasitoid interactions (Stenberg *et al.* 2007), mainly involving three closely related species (*G. calmariensis*, *G. pusilla*, and *G. tenella*) with similar life cycles and their shared wasp parasitoid (*Asecodes parviclava*) (Hambäck *et al.* 2013; Fors *et al.* 2016). The divergence of these three species is fairly recent: *G. pusilla* and *G. calmariensis* diverged around 77,000 years ago while *G. tenella* diverged around 400,000 years ago (Hambäck *et al.* 2013). *G. pusilla* and *G. calmariensis* share an exclusive host plant (*L. salicaria*), whereas *G. tenella* feeds primarily on *Filipendula ulmaria* and occasionally on other Rosaceae species. In all three species, adults in the study area overwinter until mid-May and then lay eggs on leaves or stems of their host plant. Larvae hatch after 1–2 weeks, pupate in late June to early July, and the adults emerge from the pupae by the end of July. The three species are attacked by the same endoparasitoid wasp *A. parviclava*, which lays one or more eggs in the beetle larvae. The successful wasp larvae kills the host, use it as food resources, and subsequently emerge during the following summer (Hansson and Hambäck 2013).

The demography, host searching behavior, and immunology have previously been addressed in several *Galerucella* species (Zheng *et al.* 2008; Fors *et al.* 2015; Yang *et al.* 2020), but no genome assemblies of *Galerucella* species are currently available. The closest related species that has an available genome assembly is ragweed leaf beetle (*Ophraella communa*), which also belongs to the leaf beetle (Chrysomelidae) family (Bouchemousse *et al.* 2020). Here, we report *de novo* genome assemblies for *G. calmariensis*, *G. pusilla*, and *G. tenella*. We performed computational annotation, assigned gene ontology to functional proteins, and performed ortholog cluster analysis between the three species. These draft genomes will be useful for understanding the mechanisms underlying beetle interactions with parasitoid and plant use, and for future population genomics studies (McKenna 2018).

## Materials and methods

### DNA extraction and sequencing

Larvae samples of *G. pusilla* and *G. calmariensis* were collected in mid-May 2018, from Iggön (59° 2'30.81″N, 17° 9'49.35″O), Sweden. *G. tenella* samples were collected in mid-May 2018, from Södersjön (59°51'8.72″N, 18° 6'26.59″O), Sweden. To reduce heterozygosity and bacterial contamination, we reared and inbred the beetles in the laboratory at room temperature for one generation and collected adults from the second generation for DNA extraction.

For each species, we extracted DNA from one individual using an adjusted version of the 10X Genomics sample preparation protocol “DNA Extraction from Single Insects” (https://assets.ctfassets.net/an68im79xiti/3oGwQ5kl6UyCocGgmoWQie/768ae48be4f99b1f984e21e409e801fd/CG000145_SamplePrepDemonstratedProtocol_-DNAExtractionSingleInsects.pdf). DNA concentrations were measured with a Qubit 3.0 Fluorometer using the dsDNA HS Assay Kit (Thermo Fisher Scientific) and DNA integrity was assessed on an agarose gel stained with 2% GelRed. 10X Genomics Chromium linked-read sequencing libraries were prepared and subsequently sequenced to yield paired-end 2x150 bp reads, on a HiSeq X platform at SciLifeLab (Stockholm, Sweden).

### Genome assembly and scaffolding

Raw 10X genomics reads were checked for sequencing quality using FastQC v0.11.5 (Andrews 2010), and *de novo* assembled using the Supernova v2.1.0 (Weisenfeld *et al.* 2017) assembler. We then polished the draft assembly using purge_dups v1.0.1 (Guan *et al.* 2020) to remove haplotigs and heterozygous overlaps based on sequence similarity and read depth. Subsequently, assemblies were scaffolded using arcs v1.0.6 (Yeo *et al.* 2018) and links v1.8.6, with the -a parameter, which controls the maximum link ratio between two best contig pairs set to 0.7 (Warren *et al.* 2015). To remove sequence contamination from the assembly, we ran Kraken v2.0 (Wood and Salzberg 2014) against bacterial, archaeal, and viral domains, along with the human genome. We assessed the completeness of our polished genome assemblies assessed by Benchmarking Universal Single-Copy Orthologs v4.0.5 (BUSCO) (Simão *et al.* 2015) from OrthoDB v9.1 (Zdobnov *et al.* 2017) using Endopterygota as the taxonomic database.

### Gene annotation and phylogenetic analysis

We first assessed the repeat content of our genome assemblies and created a specific repeat library using RepeatModeler v1.0.11 (Smit *et al.* 2010b) for each genome assembly. Based on the repeat library, identification of repeat sequences in the genome was performed using RepeatMasker v3.0.9 (Smit *et al.* 2010a) and RepeatRunner (Yandell 2006) with default settings. RepeatRunner is a program that integrates RepeatMasker with BLASTX (Altschul *et al.* 1997), allowing the analysis of highly divergent repeats and identifications of divergent protein-coding portions of retro-elements and retroviruses.

Gene annotation was performed using the MAKER package v3.01.02 (Holt and Yandell 2011). First, for each genome, we generated one initial evidence-based annotation using both protein and transcriptome data sources. Protein databases came from the UniProt Swiss-Prot database (downloaded on 2019-11; 561,356 proteins) (Engler *et al.* 2020), as well a subset of manually selected proteins (uniport request: taxonomy: “Coleoptera [7041],” existence: “Inferred from homology [3],” 161,853 proteins). In addition to protein resources, transcriptome data containing 57,255 transcripts from *G. pusilla* were used as evidence for all three genomes (Yang *et al.* 2020). Next, we used the candidate genes from the initial annotation to train two different *ab initio* gene predictors: Augustus v3.3.3 (Stanke *et al.* 2006) and Snap v2013_11_29 (Korf 2004). Finally, an *ab initio* evidence-driven gene build was generated based on the initial evidence-based annotation and the *ab initio* predictions. In addition, we used EVidenceModeler v1.1.1 (Haas *et al.* 2008), which allows the construction of gene models based on the best possible set of exons produced by the *ab initio* tools, and chooses those most consistent with the evidence. Functional inference for genes and transcripts was performed using the translated CDS features of each coding transcript.

Each predicted protein sequence was run against InterProscan (Jones *et al.* 2014) in order to retrieve functional information from 20 different sources. In addition, Blastp v2.9.0 (Altschul *et al.* 1990) was performed against the complete Swiss-Prot/UniProt database (downloaded 2019–2011) with a maximum *e*-value cut-off of 1_*e*_-6 to assign putative functions to predicted proteins. tRNA has been predicted through tRNAscan v1.3.1 (Lowe and Eddy 1997).

To confirm the evolutionary relationships between the three *Galerucella* species and their position in the Chrysomelidae family, we reconstructed a species tree based on predicted protein sets from *G. calmariensis*, *G. pusilla*, and *G. tenella, O. communa*, and *L. decemlineata* using OrthoFinder v2.4.0, with default settings except using multiple sequence alignments (-M msa) to infer the species tree (Emms and Kelly 2015).

### Ortholog cluster analysis

Identifying shared orthologous clusters allows the comparison of the function and evolution of proteins across closely related species. An ortholog cluster analysis was performed by comparing the three complete *Galerucella* protein sets with each other via OrthoVenn2 (Xu *et al.* 2019) with default settings of E = 1_*e*_-5 and an inflation value of 1.5.

### Data availability

Raw read data and final assemblies are available at the EMBL-ENA database under BioProject PRJEB44256. Supplementary material including annotations is available at figshare: https://doi.org/10.6084/m9.figshare.c.5470650. Command-line arguments and scripts for this study are available at: https://github.com/Pikayy/Galerucella.

## Results and discussion

### Genome assemblies

Sequencing of the 10X genomics libraries yielded a total of 683.34 million read pairs, resulting in a sequencing depth above 110X for each species. Due to the low molecular weight of the input DNA (average size <20 kbp), the initial *de novo* assembly from Supernova was highly fragmented, with N50 values of 49.884 kbp, 19.764 kbp, and 24.604 kbp for *G. calmariensis*, *G. pusilla*, and *G. tenella*. Redundancy removal by purge_dups and arcs+links scaffolding dramatically improved N50 values of assemblies (see Supplementary Table S1 for the comparisons between assemblies). The decontamination process removed two contigs from *G. calmariensis*, one contig from *G. pusilla* and two contigs from *G. tenella* which matched the human database with a kmer length >100 bp. Final assemblies for *G. calmariensis* had a size = 588 Mbp, contained 39,255 scaffolds with a N50 = 79.674 kbp, final assemblies for *G. pusilla* had a size = 513 Mbp, 40,929 scaffolds with a N50 = 45,442 kbp whereas final assemblies for *G. tenella* has a size = 460 Mbp, 29,202 scaffolds with a N50 = 31,588 kbp (Table 1). Using 2124 BUSCO groups with endopterygota_odb10 database, we found 91.3% complete orthologs and only 4.0% missing orthologs in *G. calmariensis*, 85.3% complete orthologs and 6.5% missing orthologs for *G. pusilla* and 95.4% complete orthologs and 3.3% missing orthologs for *G. tenella*. Although the final assembly was still fragmented, the completeness of genome measured by BUSCO was satisfactory. The GC content of the three genomes ranged from 33.6 to 33.8%, which is slightly higher than the GC content of the ragweed leaf beetle genome assembly (Bouchemousse *et al.* 2020).

**Table 1:** Summary of *G. calmariensis*, *G. pusilla*, and *G. tenella* reference genomes

Species	Assembly size (Mbp)	Number of scaffolds	Scaffold N50 (kbp)	Max scaffold length (Mbp)	Number of *Ns*	GC (%)	BUSCO (Complete %)
*G. calmariensis*	588.27	39,255	79.674	1.307	37,346,600	33.8	1941/91.3
*G. pusilla*	513.24	40,929	45.442	3.034	36,426,200	33.7	1812/85.3
*G. tenella*	460.59	29,202	31.588	0.234	2,669,407	33.6	2027/95.4

BUSCO score is based on the Endopterygota_db10 dataset.

The sizes of the genome assemblies of our three *Galerucella* species varied (460 to 588 Mbp) but is slightly smaller than the size of the colorado potato beetle (*Leptinotarsa decemlineata*) (642 Mbp) (Schoville *et al*. 2018) and the ragweed leaf beetle (774 Mbp) (Bouchemousse *et al.* 2020). Coleoptera is amongst the most diverse insect orders in terms of genome size, with an average genome size of 760 Mbp and ranges from 160 to 5020 Mbp (Gregory 2021). Within-genus variation in genome size is relatively small in these three assemblies compared with other Coleopteran species, possibly because of their close phylogenetic relationships and similarities in life cycle, food sources, and wasp enemies.

### Gene annotation and phylogenetic analysis

RepeatMasker masked 48.55, 46.65, and 40.84% of the *G. calmariensis*, *G. pusilla*, and *G. tenella* genomes as repetitive elements. In addition, RepeatRunner further masked approximately 1% of each genome as repeats using MAKER TE as library (Supplementary Table S2).

The *ab initio* evidence-driven annotation using the MAKER pipeline revealed 32,294, 30,302, and 33,794 potential protein-coding genes, accounting for 16.2, 17.7, and 19.1% of the whole genome of *G. calmariensis*, *G. pusilla*, and *G. tenella* respectively (Supplementary Table S3). For each species, 84 to 86% of protein-coding genes were assigned with a putative function, and 39–45% had a GO annotation (Supplementary Table S4, functional annotations using InterProscan from 20 different sources). Blast against the UniProt/Swiss-Prot database predicted 15,046, 14,404, and 17,958 hits with unique gene names for *G. calmariensis*, *G. pusilla*, and *G. tenella*, respectively.

**Figure 1:**
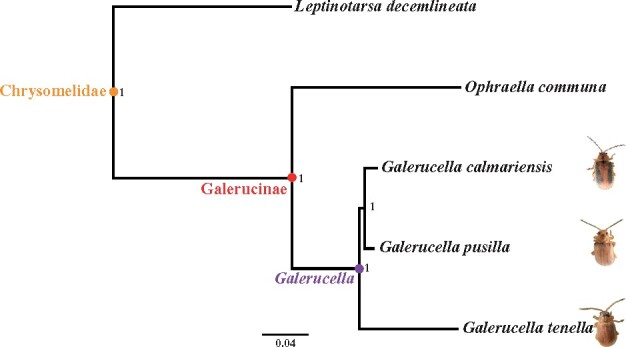
Rooted phylogenetic species tree of *G. calmariensis*, *G. pusilla*, *G. tenella*, and two leaf beetle species, *O. communa* and *L. decemlineata* (outgroup), derived from 1242 orthogroups using OrthoFinder. Branch labels indicate support values based on 1000 bootstrap replicates.

A maximum likelihood tree was built based on 1242 orthogroups shared between three *Galerucella* species and two other leaf beetles (Figure 1). The phylogenetic relationship is in accordance with previous studies generated by mitochondrial and nuclear genetic markers with high bootstrap support values for each branch (Hambäck *et al.* 2013; Bouchemousse *et al.* 2020).

### Ortholog cluster analysis

**Figure 2:**
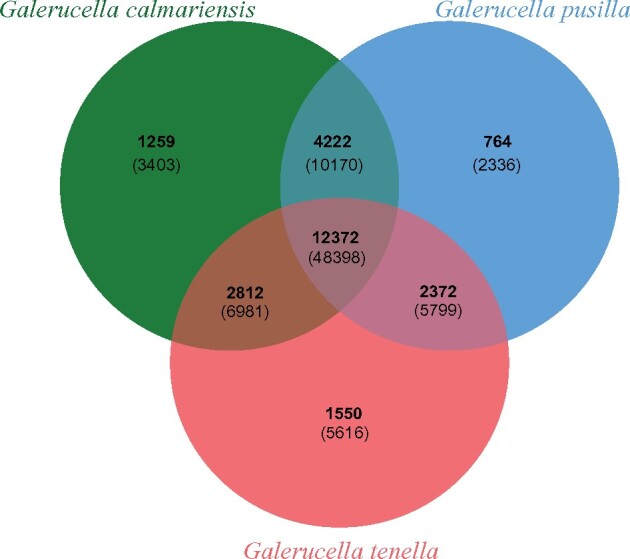
A Venn diagram of the orthologous gene clusters among the three *Galerucella* species: *G. calmariensis*, *G. pusilla*, and *G. tenella*. The numbers of shared Ortholog clusters between species is indicated in the overlapping areas of the circles while the numbers of proteins corresponding to each cluster are underneath in parentheses.

The three protein sets of *Galerucella* species were compared to identify shared orthologous clusters using OrthoVenn2 (Figure 2). The complete protein sets contain 40,031 sequences from *G. calmariensis*, 37,514 sequences from *G. pusilla* and 44,200 sequences from *G. tenella*, corresponding to 20,665, 19,730, and 19,106 ortholog clusters, respectively.

Most annotated genes (12,372 orthogroups/48,398 proteins) were shared between the three species. Shared clusters between *G. calmariensis* and *G. pusilla* (16,594) account for 80.3 and 84.1% of ortholog clusters in *G. calmariensis* and *G. pusilla* respectively whereas the shared regions of either *G. calmariensis* and *G. pusilla* with *G. tenella* account for less than 75% of their clusters. Ortholog clusters unique to a single species account for 6.09, 3.87, and 8.11% of the entire cluster set for *G. calmariensis*, *G. pusilla*, and *G. tenella*, which indicates divergent regions between species (Ferguson *et al.* 2020). The inflated numbers of singleton clusters in *G. tenella* may be due to the high duplication levels in the genome, as BUSCO detected 33.1% complete duplicated BUSCOs in *G. tenella*. Whether this is due to gene duplication or assembly error should be further investigated. The duplication and fragmentation level detected by BUSCO are similar between *G. calmariensis* (1.8% duplicated and 5.1% fragmented) and *G. pusilla* (1.2% duplicated and 8.2% fragmented), however, *G. calmariensis* harbors a higher level of singleton clusters than *G. pusilla*.

## Conclusions

*Galerucella* species play an important role as biological control agents as well as for ecological and evolutionary research. Here, we produced draft genome assemblies for three leaf beetles in the *Galerucella* genus, which are the first three genomes from the Galerucinae subfamily branch of the leaf beetle family. The genome sequencing of the three closely related beetles sharing a common wasp enemy also provides possibilities of understandings of food web and host-parasitoid interactions. In particular, comparing genomes of species with divergent immune resistance against parasitoid wasps may contribute to detecting essential genetic regions underlying host immunity and other potential traits participating in the arms race between host and parasitoids.
